# The Otranto Channel (South Adriatic Sea), a hot-spot area of plankton biodiversity: pelagic polychaetes

**DOI:** 10.1038/s41598-019-55946-6

**Published:** 2019-12-20

**Authors:** Rosanna Guglielmo, Alessandro Bergamasco, Roberta Minutoli, Francesco P. Patti, Genuario Belmonte, Nunziacarla Spanò, Giacomo Zagami, Vincenzo Bonanzinga, Letterio Guglielmo, Antonia Granata

**Affiliations:** 1 SZN- Stazione Zoologica Anton Dohrn, Villa Comunale, 80121 Napoli, Italy; 2 CNR-ISMAR (Institute of Marine Sciences, National Research Council), Arsenale - Tesa 104, Castello 2737/F, 30122 Venice, Italy; 3 Dipartimento di Scienze Biologiche, Chimiche, Farmaceutiche ed Ambientali, Università di Messina, 98100 Messina, Italy; 4 CoNISMA O.U. Lecce, DiSTeBA-University of Salento, 73100 Lecce, Italy; 5 Dipartimento di Scienze biomediche, odontoiatriche e delle immagini morfologiche e funzionali, Università di Messina, 98100 Messina, Italy

**Keywords:** Biooceanography, Biodiversity

## Abstract

Composition, density and specimen sizes of pelagic polychaete assemblages were analyzed in the Southern Adriatic Sea. The study was based on finely stratified vertical (0–1100 m) and spatial sampling (17 stations) representing spring conditions. Holoplanktonic polychaetes were distributed in both neritic and pelagic waters, although the highest densities were observed along the Otranto Channel. Analysis of the size frequency distribution revealed a trend with depth only for some species. Spatial distribution of holoplanktonic polychaete density was not related to bottom depth, being the organisms mainly concentrated in the epipelagic layer (0–100 m). The most abundant species showed maximum values below or within the thermocline and within the Deep Chlorophyll Maximum or just above it. Relations between polychaete presence and the underlying oceanographic mechanisms regulating the circulation in the Otranto Channel were discussed. The presence of several non-determined polychaete larvae (e.g. Syllidae) in the pelagic waters at 800–1100 m depths suggests the importance of the role of Levantine waters as main actual and potential carrier of species in the area, though a relevant contribution comes also from North Adriatic dense waters through deep spilling and cascading in the Southern Adriatic pit. These findings increase the knowledge on holoplanktonic polychaetes ecology within the South Adriatic Sea, and represent significant data in the monitoring of changes in biodiversity.

## Introduction

Polychaetes are a large and diversified group, widely distributed in freshwater and marine habitats, from the intertidal zone to the deepest sediments. There are about 9000 nominal polychaete species in the world^1^, but their diversity is supposed to be much larger and there are many species that have yet to be described. Most of them inhabit the benthic environment, although several species and a few families have evolved to colonize the pelagic realm, showing hyaline body and large appendages for floating and swimming^2–4^.

Holoplanktonic polychaetes are common in marine zooplankton^3^, although they are not highly important in terms of abundance^5^. Some species may, at times, be the dominant forms in the plankton community^6–8^. They are widely distributed in the oceans, mainly in the open sea, but they also occur in neritic region^3^. Pelagic polychaetes inhabit the entire water column, most species are found in the 100 m thick surface layer^9^, even if several forms have bathypelagic habits^10^.

In spite of their low abundance in the plankton, but in consideration of a wide range of feeding strategies, holoplanktonic polychaetes play an important role in the pelagic food web^11,12^ and in organic matter mineralization^13^. Their trophic ecology is complex: (i) most species are active predators and use their quickly eversible proboscis to attack other zooplankters, such as fish larvae, siphonophores, chaetognaths and appendicularians (ii) some others are filter-feeders or phytophagous and (iii) only few species are detritivorous^13^. In turn, they are a basic food, rich in calories, of numerous fishes^3^ and of predatory large copepods^7^.

Most studies on holoplanktonic polychaetes have emphasized taxonomy, but ecological aspects are poorly known^12^. Spatial distribution of the families Typhloscolecidae and Tomopteridae in the tropical eastern part of the Pacific Ocean is available^14,15^ as a first step towards understanding the pelagic polychaete fauna in the tropical western Atlantic region^16^. Many authors have demonstrated a strong correlation between the distribution of planktonic polychaetes and water masses circulation in the South Atlantic and North Pacific^17–19^, or suggested that the structure of holoplanktonic polychaete assemblage (oceanic and neritic) could be determined by the feeding habits of the species and their tolerance to the variability in environmental conditions^12^.

As regards Mediterranean Sea, pelagic polychaetes have been largely neglected respect to the benthic polychaete fauna. Monthly changes in composition and abundance of meroplankton and pelagic polychaetes (6 species) of the Cilician Basin shelf waters were reported^20^, whereas in the checklist of Anellida Polychaeta of the Eastern Mediterranean Sea regions, 14 species of planktonic polychaetes were included from the coasts of Turkey^21^. More recently, two species of both Typhoscolecidae (*Sagitella kowalewski* Wagner, 1872 and *Typhloscolex muelleri* Busch, 1851) and Iospilidae (*Phalacrophorus pictus* Greeff, 1879 and *Iospilus phalachroides* Viguier, 1886), already included in the checklist of polychaetes of the Italian seas^22^, have been reported for the first time in superficial waters (20–50 m depth) along the northwestern coast of Isola d’Elba (Tuscany)^23^.

The first list of all pelagic and benthic polychaetes of the Adriatic Sea was compiled in early nineties^24^ and included 559 species belonging to 53 families. As for the holoplanktonic polychaetes it is based on several contributions during the last century^25–32^. Compared with earlier studies in the Southern Adriatic Sea, many papers presented data on participation of polychaetes in total zooplankton^9,33–35^ focusing the attention on the temporal variation of the abundances of some species of polychaetes in order to hypothesize faunal changes associated with the changes of pelagic waters circulation in the South Adriatic through the Otranto channel and on pelagic polychaete distribution and seasonal dynamics^36^. More recently, a complete polychaete checklist of the whole Adriatic Sea was compiled^37^, in which 24 species of holoplanktonic polychaetes were reported. However, information on the vertical distribution of the holoplanktonic polychaetes population in the different hydrographically defined sub-basins remains lacking.

This study aims at describing the composition and abundance patterns of the holoplanktonic polychaete communities in the Southern Adriatic waters, across the Otranto Channel key area and contributes to fill some knowledge gaps thanks to a finely stratified spatial samplings up to 1100 m depth. The general objectives are to shed light on (1) how the pelagic polychaete distribution and size patterns link to environmental conditions and variability such as horizontal gradients and vertical structure of the water column and (2) how the meso-scale oceanographic circulation can modulate the Northern Adriatic and Ionian inputs of species in the Southern Adriatic Basin, through climate-regulated mechanisms.

## Methods

### Study area

The study area includes the Southern Adriatic basin (maximum depth 1200 m), the Otranto Channel (lat 40°N, lon 19°E, 70 km wide) and its interface with the Ionian Sea, a key region for the regulation of the deep overturning cell of the eastern Mediterranean basin^38^. In particular, the influence of the Adriatic Deep Water (ADW) outflow through the Otranto Channel triggered by the formation of dense waters in the Adriatic basin during winter intense cooling events has been put in connection with the periodic reversal of the main upper layer circulation in the Ionian Sea and the consequent alternate advection of Atlantic or Levantine waters in the Southern Adriatic basin (the BIoS mechanism)^39^. Moreover open-ocean deep convection can be responsible during winter for the production of dense water, generating a mixture of the less saline waters from the Adriatic Sea with the more saline and warmer waters originating from the Ionian Sea^40^.

The role of the Adriatic Sea as the main source of dense water is known to strongly depend upon the atmospheric and thermohaline conditions^41^. In fact, in the early 1990s the Aegean Sea became the main deep-water formation area (the event is known as the Eastern Mediterranean Transient-EMT), with relevant implications for the water mass properties and circulation of the whole levantine basins^42,43^. The mechanism seems nowadays re-established and the Adriatic Sea is returning to dominate the eastern deep-water production^44^.

The surface layer of the Southern Adriatic basin is characterized by the presence of Adriatic Surface Water (ASW), that mainly flows geostrophically along the Italian coast and features a relatively lower salinity due to the freshwater discharges (Po river) and the Ionian Surface Water (ISW), saltier (S > 38.25) and warmer (T > 15 °C) than ASW, that flows into the Adriatic along the eastern side of the Otranto Channel. During winter, the ISW is spread by the dominating south-easternly winds and in spring it occupies almost the whole basin. Through the same gateway along the eastern border the Levantine Intermediate Waters (LIWs) flow into the Southern Adriatic basin where they undergo local mixing and form the Modified Levantine Intermediate Waters (MLIWs), characterized by salinity maximum. MLIWs are defined by S > 38.6 and T > 13.5°C and in the Southern Adriatic basin they can be identified in the layer 150–400 m^45^. The impact of LIW inflow on the biogeochemical cycles in the Adriatic Sea is substantial, and the fluctuation of a number of physical, chemical, and biological parameters in the Adriatic Sea has been attributed to the LIW ingression^9,46,47^. In the deepmost layer of the Southern Adriatic basin the Adriatic Deep Water (AdDW) can be found. According to density, two types of AdDW can be distinguished: the North Adriatic Deep Water (NAdDW, σ_t_ > 29.2), the newly formed dense water that can reach in 2–3 months directly the Southern basin by flowing along the Italian coast or spilling through the Central Adriatic Jabuka Pit^48^ and the Southern Adriatic Deep Water (SAdDW, σ_t_ > 29.1), considerably warmer and saltier than NAdDW, that forms in the Southern basin by mixing with the overlaying MLIW.

### Sampling procedure

**Figure 1:**
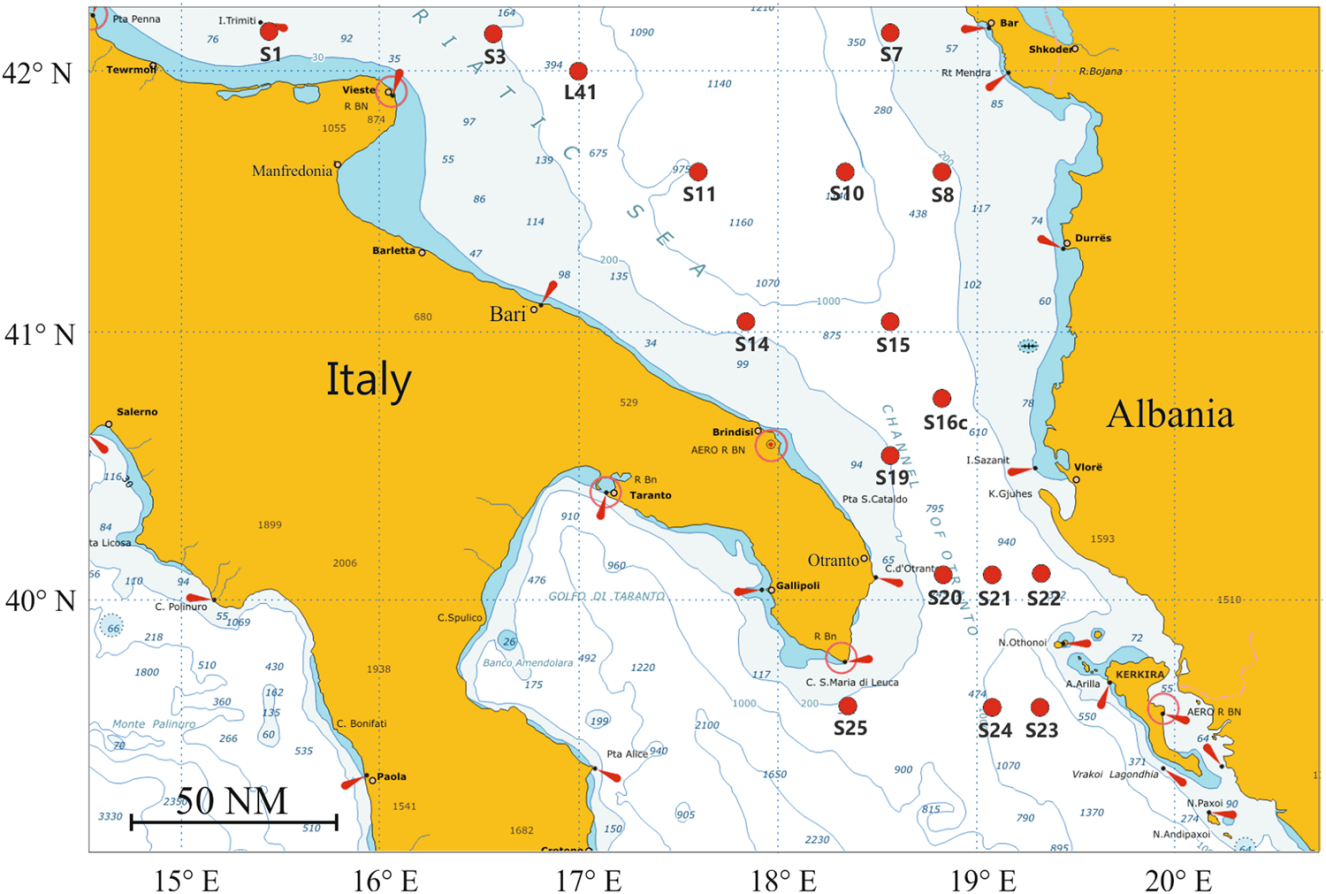
South Adriatic Sea: BIONESS sampling stations during the CoCoNet-WP11 oceanographic cruise, May 8−21, 2013.

The examined zooplankton samples were collected during the CoCoNet multidisciplinary oceanographic cruise carried out from 8 to 21 May, 2013 in the Southern Adriatic Sea by the R/V Urania. The sampling included 17 stations (Table 1) on the Apulian and Albanian shelves and offshore waters, including the Strait of Otranto (Fig. 1). Vertical profiles of temperature and salinity were collected in each stations with a SBE19 CTD (Sea-Bird Scientific, USA) to describe the overall oceanographic context of zooplankton samples. A total of 146 zooplankton samples were collected in several layers between the surface and few meters above the seabed, along a 0–1100 m water column. Samples were measured with the electronic multinet EZ-NET BIONESS (Bedford Institute of Oceanography Net Environmental Sampling System)^49^. This 0.25 m^2^ mouth model was equipped with 10 nets (mesh size 230 μm) and with a multi-parametric probe SBE 911plus (Sea-Bird Scientific, USA), which continuously recorded temperature, salinity, towing depth and fluorescence. Initial real-time data on depth (m), temperature (°C), salinity and fluorescence (μg L^−1^ Chl *a*) were processed with Ocean Data View (ODV) software^50^ to obtain vertical profiles. Flow velocity and filtration efficiency were monitored by two internal and external flowmeters. The BIONESS was deployed at low speed along an oblique path to the maximum selected depth to be investigated, towed at a speed of 1.5–2 m s^−1^ until closing, with the simultaneous opening of a new net. The nets were opened and closed on command from the ship at selected sampling layers according to vertical profiles of associated biological and physical parameters. The filtered volume of water in each layer varied between 15 and 107 m^3^, according to the thickness of the sampled layer (Table S1). The number and the thickness of the sampled strata depended on the bottom depth. In the uppermost 100 m depth, the sampled layers were 10–40 m of thickness, and between 100 and 1300 m the layer thickness increased to 50 and 300 m. On board, the samples were preserved in 4% buffered formaldehyde seawater solution.

**Table 1:** CoCoNet-WP11 May 2013 cruise in the South Adriatic Sea. Stations and sampling dates.

Station	Local date	Position		Local Time		Bottom depth	Max sampled depth
		Lat. N	Long. E	Start	End	(m)	(m)
S1	9-May-2013	42°09.994′	15°39.966′	20:23	21:37	99	90
L41	10-May-2013	41°59.952′	16°59.872′	18:38	20:21	580	550
S3	10-May-2013	42°09.985′	16°38.059′	14:05	15:47	178	170
S10	11-May-2013	41°29.780′	18°22.453′	23:48	02:07	1123	1096
S7	11-May-2013	42°10.049′	18°32.310′	15:28	16:46	190	180
S8	12-May-2013	41°29.971′	18°50.127′	04:47	06:35	324	310
S16c	13-May-2013	40°53.072′	18°57.210′	11:48	13:09	317	300
S15	13-May-2013	41°02.365′	18°31.554′	05:36	07:38	939	900
S22	14-May-2013	40°05.222′	19°21.708′	18:03	20:25	965	900
S21	14-May-2013	40°05.008′	19°08.001′	22:17	00:28	972	900
S23	15-May-2013	39°40.001′	19°22.009′	18:01	20:29	1172	1100
S24	15-May-2013	39°40.004′	19°08.008′	22:14	00:23	1089	1000
S20	16-May-2013	40°05.012′	18°50.071′	14:50	16:41	738	700
S25	16-May-2013	39°39.917′	18°22.140′	04:26	05:59	261	210
S19	17-May-2013	40°26.801′	18°32.195′	10:19	11:42	127	100
S14	17-May-2013	41°02.305′	17°52.030′	18:02	19:51	699	600
S11	18-May-2013	41°29.991′	17°34.972′	07:10	09:25	1137	1060

Stations and sampling data.

### Laboratory zooplankton analysis

In the laboratory, the pelagic polychaetes were sorted out from the total sample, identified, and counted under a Leica Wild M10 stereomicroscope. Adult pelagic polychaetes were counted and identified to the lowest possible taxonomic level following the available descriptions^3,51–57^. Differently from all the members of the family Tomopteridae, Alciopidae are mostly very delicate, slender animals, and are rarely found complete; most of them may still be identified from a head fragment. Therefore, it is in most cases impossible to indicate dimensions and number of segments, but Apstein^58^ has given a key to the genera by which we may determine a specimen from only one well-preserved parapodium. This key forms also the base of Fauvel’s key^51^ and subsequently other authors^55,59,60^. The polychaete larvae have been identified at family level (Spionidae) or nondetermined. Polychaetes vertical density distribution was estimated from the total specimens counted in any sampled layer divided by the volume of the filtered water and expressed as number of individuals per 100 m^3^ (ind.100 m^-3^).

In this paper, we present standing crop estimates by a weighted mean of pooled layers between 0 and 200 m, standardized as number of individuals per m^2^. Randomly selected specimens of abundant complete polychaete species encountered in each sampled stratum were measured for Total Length (TL, from prostomium to pygidium) to the nearest millimeter, to obtain minimum–maximum length range and mean length for each species^61^. Shrinkage due to formaldehyde preservation (ca. 10%) was not considered. A more detailed study was conducted on the vertical distribution of the three species that dominated the polychaetes community.

Polychaete abundance and TL patterns across the relevant factors (station, layer depth, habitat type along the column, water mass type) and the main environmental variables (temperature, salinity and chlorophyll) were studied by using R tool packages and the StatGraphics software packages. To test for significant differences between and within groups of data, analysis of variance (one-way ANOVA) was employed, coupled with parametric (Fisher’s LSD on means, in case of homogeneity of variances) or non-parametric (Kruskal-Wallis on medians) tests. The non-parametric Spearman rank correlation test has been used to measure the degree of association between two variables. Moreover, distance-based Redundancy Analysis (dbRDA) was used to describe similarity patterns in holoplanktonic polichaetes communities associated with environmental parameters (latitude/longitude, depth, temperature, salinity and chlorophyll *a*). Bray-Curtis distance applied to non empty samples was used. All analyses were performed using ‘vegan’ package in R. Sample-based abundance data were used to estimate overall asymptotic richness in the dataset and rarefaction/extrapolation of species richness in the reference samples of the different water mass types through the software package EstimateS 9.10^62^. Expected species richness was calculated through the Chao1 estimator (classic formula) and first order jackknife analysis. Completeness of the samples was evaluated according to the approach proposed by Chao & Jost^63^.

## Results

### Environmental parameters

The data by the BIONESS were used to define the spatial variability of environmental parameters (T, S and Chl *a*) during the spring cruise (CoCoNet 2013) in the Southern Adriatic Sea. Horizontal and vertical variability of thermohaline characteristics in the area evidenced some marked differences both among stations and along the sampled water column.

**Figure 2:**
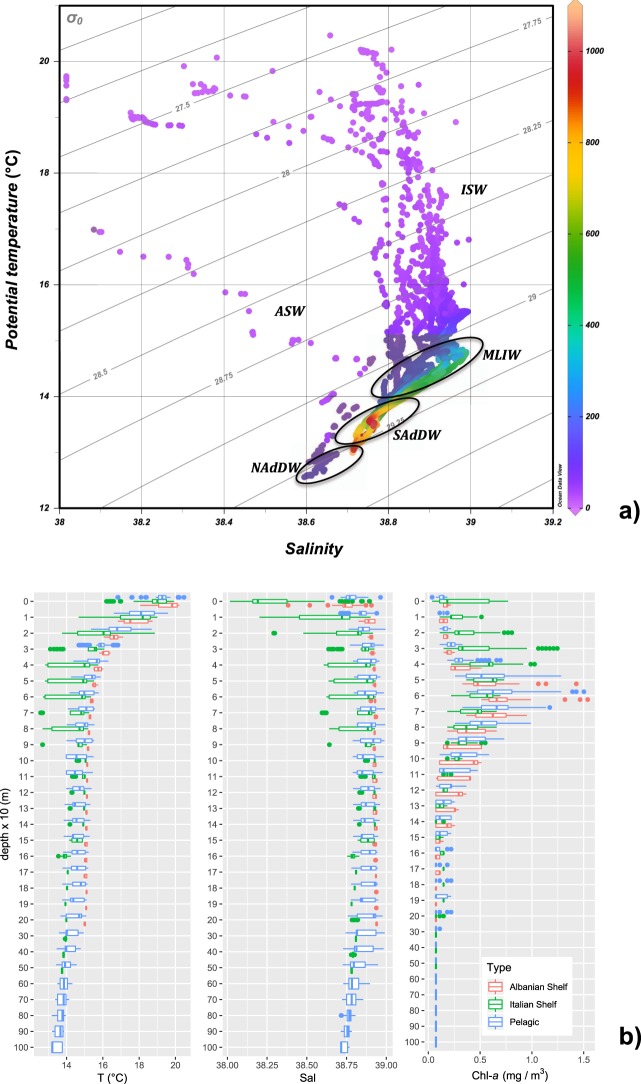
Oceanographic context during the CoCoNet Cruise in Southern Adriatic Sea (May 2013). (**a**) θ-S diagram of the collected CTD profiles. Colors indicate the depth (m). Water mass types are highlighted. (**b**) Vertical profiles of temperature (°C), salinity and chlorophyll *a* (µg/L) from all the BIONESS sampled stations. Vertical scale is expanded in the 0−200 m layer (where original data are pooled every 10 m) with respect the remaining of the water column (where original data are pooled every 100 m).

The Τ-S profiles collected during the campaign by a SBE19 CTD are shown in Fig. 2a. Mid May conditions well represent the middle phase of spring in Southern Adriatic, with a different water masses vertical structure among Apulian, offshore and Albanian stations. In particular, the upper layer up to 100–150 m is dominated by ISW except along the Italian coast, where ASW can be found. The intermediate layer hosts the MLIW and the deep layer is occupied by ADW originated in the Southern basin (SAdDW) or formed in the Northern basin (NAdDW). According to this structure, the 146 BIONESS samples were labeled to form 4 clusters: 5 samples were assigned to ASW, 71 to ISW, 28 to MLIW and 42 to ADW. Among these latter, 8 were assigned to NADdW and 34 to SAdDW.

T-S BIONESS profiles provide an overall hydrographical picture of the all investigated area (Fig. 2b), showing an evident stratified temperature and a marked thermocline that separates the upper layer from the underlaying layers. On average, the Italian side was occupied by colder and less salty waters than Albanian coast. Surface temperature showed increasing trends from Italian (T = 18.90°C, S = 38.85) to Albanian (T = 20.20°C, S = 38.94) side, determined by less salty water masses from Northern Adriatic. A shallow thermocline and a colder temperature were recorded on the Italian side (St. 14, 19; 30–50 m; 15.8–14.5 °C) rather than in the Albanian one (St. 7, 8; 30–65 m; 16.1–15.5 °C). In the pelagic area (St. 15, 16c) the thermocline is deeper (60–90 m) with an average temperature between 15.2 and 14.9°C. In the Otranto Channel (St. 20, 21, 22) the thermocline was recorded between 32 and 65 m, with an average temperature between 16.0 and 15.4°C. In the Ionian waters, at the entrance to the Otranto Channel (St. 23, 24, 25), the thermocline was between 50 and 65 m, and temperature values almost constant (15.4–15.5°C).

Chlorophyll fluorescence showed maxima in the layer between 50 m and 80 m in depth for all the stations, except for St. S1 that showed highest chlorophyll *a* concentration at about 35 m (1.17 mg m^−3^). Fluorescence profiles showed a different depth of the Deep Chlorophyll Maximum (DCM). Generally, in the areas close to the coast and in Otranto Channel this maximum was detected at 60 m, but with very different max chlorophyll *a* values: 0.696 mg m^−3^, 1.54 mg m^−3^ and 1.12 mg m^−3^, in the Apulian, Albanian side and Otranto Channel, respectively. In the offshore waters the DCM was slightly deeper (66 m, 0.72 mg m^−3^), while in the Ionian stations it was found at about 73 m (0.95 mg m^−3^). Below 120 m in depth, the chlorophyll *a* concentration dropped to very low values in all of the stations.

### Holoplanktonic polychaetes

#### Density and species composition

Mean total zooplankton density, among the 17 sampled stations, was 44,000 ind.100 m^−3^ (CV = 58%). Copepods were the most abundant taxon representing from the 72 to 96% of the total zooplankton, with a mean density of 40,000 ind.100 m^−3^ (CV = 59%), then Chaetognatha (1400 ind.100 m^−3^; CV = 113%), Ostracoda (617 ind.100 m^−3^; CV = 84%), Siphonophora (490 ind.100 m^−3^; CV = 121%) and Amphipoda (300 ind.100 m^−3^; CV = 115%) were found in decreasing order. Pelagic polychaetes represented only the 0.38% of the total zooplankton community and exhibited a mean density of 66 ind.100 m^−3^ in 102 non-empty samples (out of 146 in total).

Overall 1154 specimens were diagnosed at species level. Regarding composition, 22 species belonging to 4 families (Tomopteridae, Alciopidae, Typhloscolecidae, Lopadorrhynchidae) were identified (Table 2). Tomopteridae was the most abundant family with 44.4 ind.100 m^−3^ representing about two-thirds of total polychaete density. Among the 6 species belonging to the genus *Tomopteris*, *T. ligulata* Rosa, 1908 was the most abundant (19.4 ind.100 m^−3^) one and the most frequently observed (35% occurrence in the non-empty samples), followed by *T. pacifica* (Izuka, 1914) (7.3 ind.100 m^−3^) and *T. planktonis* Apstein, 1900 (2.5 ind.100 m^−3^). Only rare specimens of *Tomopteris apsteini* (Rosa, 1908)*, Enapteris euchaeta* Chun, 1888 and *T. catharina* (Gosse, 1853) were observed whereas juvenile specimens of this genus were diffusely present throughout the study area and reached the maximum density of 14.8 ind.100 m^−3^ (as *Tomopteris* sp.).

**Table 2:** Holoplanktonic and meroplanktonic polychaete species identified in the study area: total mean weighted  density over all stations, per cent contribution, number of counted specimens (N), size range with mean value (X), standard deviation (±SD) and number of specimens measured (n).

Species	Density (ind./100 m^3^)	Percent %	N	Size range (mm)	X (mm)	(±SD)	*n*
**Holoplanktonic**							
**Lopadorrhynchidae** Claparède, 1870							
*Lopadorrhynchus brevis* Grube, 1855	0.13	0.20	9	1.29–2.23	2.00	0.47	3
*Lopadorrhynchus uncinatus* Fauvel, 1915	0.13	0.20	9	2.11–16.47	10.37	7.48	4
*Maupasia coeca* Viguier, 1886	0.83	1.25	58	1.76–3.76	2.50	0.68	14
*Pedinosoma curtum* Reibisch, 1895	0.04	0.06	3	4.00	4.00	—	1
*Pelagobia longicirrata* Greeff, 1879	0.13	0.20	9	1.41–2.94	2.35	0.64	4
**Alciopidae** Ehlers, 1864							
*Alciopina parasitica* Claparède & Panceri, 1867	0.04	0.06	3	4.35–6.47	5.76	1.22	2
*Krohnia lepidota* (Krohn, 1845)	8.83	13.36	623	1.17–7.64	3.07	1.18	115
*Naiades cantrainii* Delle Chiaje, 1830	1.74	2.63	123	2.94–13.52	5.33	2.94	31
*Plotohelmis capitata* (Greeff, 1876)	0.07	0.10	5	6.11	6.11	—	1
*Plotohelmis tenuis* (Apstein, 1900)	0.05	0.07	3	3.17–7.64	6.15	2.58	2
*Rhynchonereella gracilis* Costa, 1864	0.37	0.56	26	1.18–5.64	4.14	1.29	6
*Rhynchonereella moebii* (Apstein, 1893)	0.05	0.07	3	9.29	9.29	—	1
*Vanadis crystallina* Greeff, 1876	2.58	3.91	182	1.17–20.58	6.57	5.16	41
*Vanadis formosa* Claparède, 1870	1.99	3.01	140	2.92–20.00	6.64	3.76	44
**Tomopteridae** Johnston, 1865							
*Enapteris euchaeta* Chun, 1888	0.05	0.07	3	12.35	12.35	—	1
*Tomopteris apsteini* (Rosa, 1908)	0.16	0.24	11	2.35–23.52	11.09	9.75	6
*Tomopteris catharina* (Gosse, 1853)	0.16	0.24	11	2.35–11.76	8.27	3.08	9
*Tomopteris ligulata* Rosa, 1908	19.41	29.38	1370	1.17–7.41	2.90	0.95	383
*Tomopteris pacifica* (Izuka, 1914)	7.28	11.03	514	1.17–7.05	3.67	1.12	154
*Tomopteris planktonis* Apstein, 1900	2.53	3.83	179	2.11–6.00	3.85	0.98	32
*Tomopteris sp.	14.79	22.38	1044	0.70–5.29	3.06	0.97	203
**Typhloscolecidae** Uljanin, 1878							
*Sagitella kowalevskii* Wagner, 1872	1.83	2.78	129	2.35–7.17	5.32	1.25	42
*Travisiopsis lanceolata* Southern, 1910	0.35	0.53	25	2.35–4.94	4.00	0.86	7
*Travisiopsis sp.	0.26	0.40	18	3.76–4.11	3.99	0.20	2
**Meroplanktonic**							
**Poecilochaetidae** Hannerz, 1956							
*Poecilochaetus serpens* larvae Allen, 1904	0.03	0.04	2	3.17	3.17	—	1
**Syllidae** Grube, 1850							
Syllidae larvae	1.18	1.78	83	2.11–3.29	2.63	0.40	13
Nondetermined polychaete larvae	1.07	1.62	76	1.76–2.58	2.17	0.30	32

*Remarks: Juvenile specimens not assigned certainly to a species.

Alciopidae ranked second in density with about 15.7 ind.100 m^−3^ (23.8% of the polychaete community). Among the 9 species identified within this family the most abundant was *Krohnia lepidota* (Krohn, 1845) (8.8 ind.100 m^−3^) occurring in 28% of the non-empty samples, followed by *Vanadis crystallina* Greeff, 1876 (2.6 ind.100 m^−3^), *Vanadis formosa* Claparède, 1870 (2.0 ind.100 m^−3^) and *Naiades contrainii* Delle Chiaje, 1830 (1.7 ind.100 m^−3^). Few specimens of the other five species (*Alciopina parasitica* Claparède & Panceri, 1867*, Plotohelmis capitata* (Greeff, 1876)*, Plotohelmis tenuis* (Apstein, 1900)*, Rhynchonereella gracilis* Costa, 1864 and *Rhynchonereella moebii* (Apstein, 1893)) together constituted only 0.20%.

Typhloscolecidae was the third family in density with 2.4 ind.100 m^−3^ (3.7% of the polychaete community). Among them *Sagitella kowalewski* was the most abundant species (1.8 ind.100 m^−3^), while *Travisiopsis lanceolata* Southern, 1910 and *Travisiopsis* sp. appeared with only few specimens. Lopadorrhynchidae family, with 1.3 ind.100 m^−3^ (1.9% of the polychaete community), was represented by 5 species with very few specimens among which *Maupasia coeca* Viguier, 1886 resulted the most abundant and frequent one. Among these species, five were recorded in the South Adriatic for the first time: *Alciopina parasitica, Pedinosoma curtum* Reibisch, 1895*, Plotohelmis capitata, Plotohelmis tenuis, Rhynchonereella moebii* (Table 3).

**Table 3:** List of holoplanktonic polychaete species reported for the Southern Adriatic Sea by this study and available literature.

Species	Southern Adriatic Sea			Synonymised names
	This Study	Mikać (2015)	Batistić *et al*. (2004, 2007, 2012)	
**Lopadorrhynchidae** Claparède, 1870				
*Lopadorrhynchus brevis* Grube, 1855	X	X		
*Lopadorrhynchus uncinatus* Fauvel, 1915	X	X		
*Maupasia coeca* Viguier, 1886	X		X	
*Pedinosoma curtum* Reibisch, 1895	X			
*Pelagobia longicirrata* Greeff, 1879	X	X	X	
**Pontodoridae** Bergström, 1914				
*Pontodora pelagica* Greeff, 1879			X	
**Iospilidae** Bergström, 1914				
*Phalacrophorus pictus* Greeff, 1879			X	
**Alciopidae** Ehlers, 1864				
*Alciopina parasitica* Claparède & Panceri, 1867	X			
*Krohnia lepidota* (Krohn, 1845)	X	X	X	=*Callizonella lepidota*
*Naiades cantrainii* Delle Chiaje, 1830	X	X		
*Plotohelmis capitata* (Greeff, 1876)	X			
*Plotohelmis tenuis* (Apstein, 1900)	X			
*Rhynchonereella gracilis* Costa, 1864	X		X	=*Callizona nasuta*
*Rhynchonereella moebii* (Apstein, 1893)	X			
*Torrea candida* (Delle Chiaje 1828)		X		
*Vanadis crystallina* Greeff, 1876	X	X	X	
*Vanadis formosa* Claparède, 1870	X	X	X	
**Tomopteridae** Johnston, 1865				
*Enapteris euchaeta* Chun, 1888	X	X		=*Tomopteris euchaeta*
*Tomopteris apsteini* (Rosa, 1908)	X	X		=*T. scolopendra*
*Tomopteris catharina* (Gosse, 1853)	X	X	X	=*T. helgolandica*
*Tomopteris cavallii* Rosa, 1908		X		
*Tomopteris ligulata* Rosa, 1908	X	X		
*Tomopteris pacifica* (Izuka, 1914)	X	X	X	=*T. elegans*
*Tomopteris planktonis* Apstein, 1900	X	X		
**Typhloscolecidae** Uljanin, 1878				
*Sagitella kowalevskii* Wagner, 1872	X	X	X	
*Travisiopsis lanceolata* Southern, 1910	X		X	
*Typhloscolex muelleri* Busch, 1851		X	X	
Number of species	22	17	13	

#### Horizontal and vertical distribution

**Figure 3:**
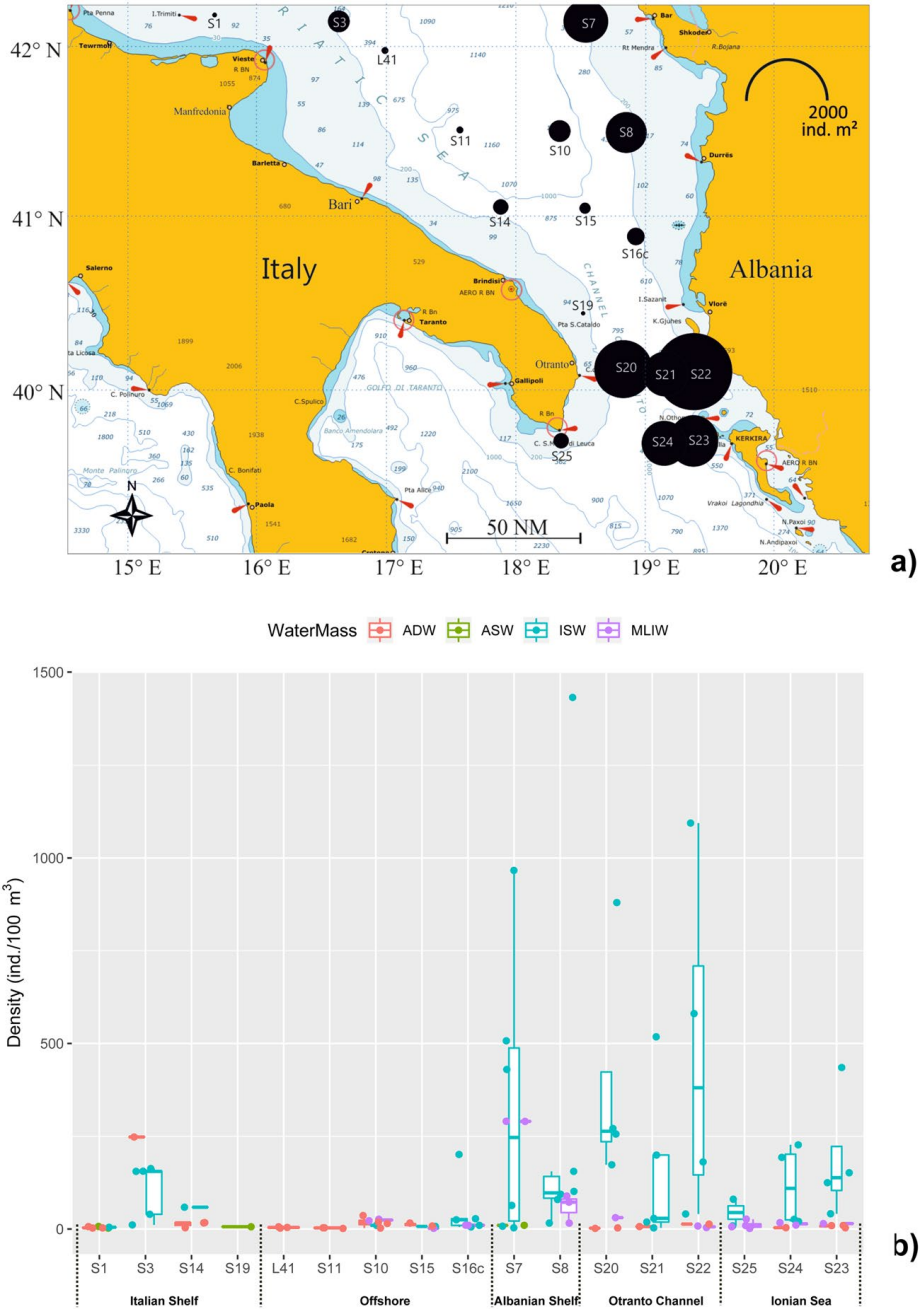
Spatial distribution of holoplanktonic polychaetes in the study area. (**a**) Total polychaete density (ind. m^−2^) integrated in the 0−200 m layer. (**b**) Polychaete density in each sample (ind. 100 m^−3^) assigned to the different water mass types present in the stations.

Holoplanktonic polychaetes were distributed in the entire study area (Fig. 3a). Most of the species were found in the 0–200 m stratum, so we considered this layer to calculate the standing crop of the entire polychaete community in the whole study area. Higher densities were recorded in the stations located in Otranto Channel, with the highest value in the Station 22 (2902 ind. m^−2^). Relatively few specimens were found along the Italian coast and in the offshore waters, while the stations along the Albanian shelf have shown higher values (Fig. 3b). This West-to-East gradient is statistically significant (ANOVA Abundances by Longitude, p < 0.001, Kruskal-Wallis) and holds even if the analysis is restricted to the 0–200 m layer, suggesting that the main part of the community occupies the upper part of the water column: in fact, density decreases with increasing depth (Spearman’s rank correlation r = −0.21, p < 0.012, N = 146).

**Figure 4:**
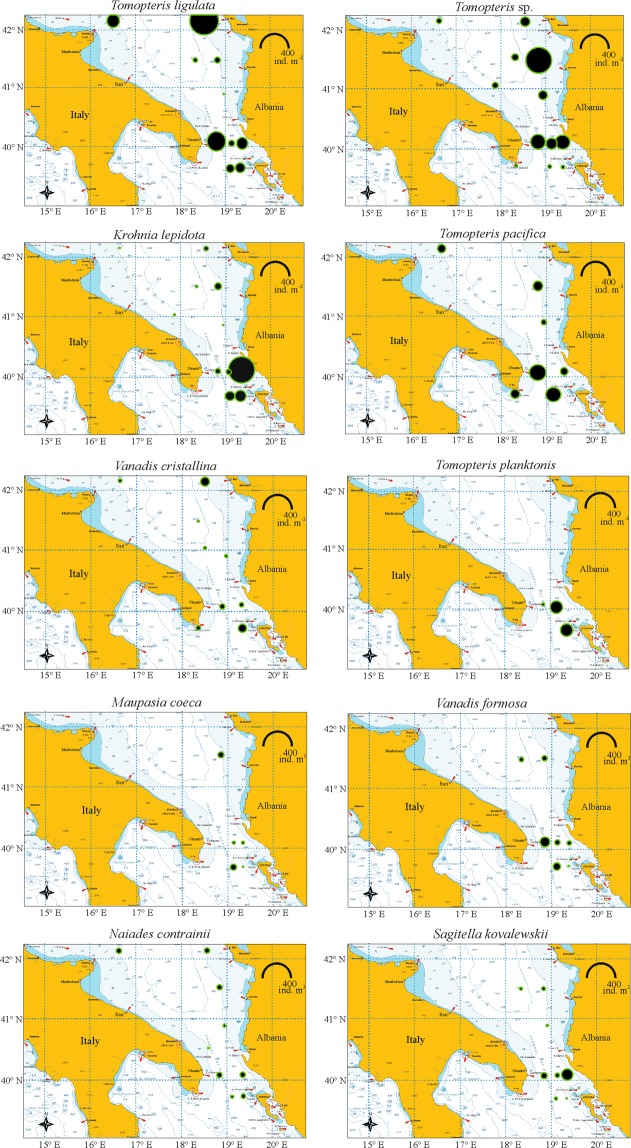
Density (ind. m^−2^) in the 0−200 m layer of the most abundant polychaete species.

The horizontal distribution of the most abundant species in the 0–200 m layer is shown in Fig. 4. *T. ligulata* showed a wide distribution (positive records in 10 stations), with highest densities along the Albanian shelf (St. S7, 408 ind. m^−2^). *Tomopteris* sp. (recorded in 12 stations) has shown maximum values in the Station S8 (338.7 ind. m^−2^), while *K. lepidota* (11 stations) exhibited the highest density in the Station S22 (347.3 ind. m^−2^), *T. pacifica* (7 stations) in Sts. S20 and S24 (124.6 and 105 ind. m^−2^ respectively), *V. crystallina* (9 stations) in Sts. S7 and S23 (39.1 and 34.8 ind. m^−2^, respectively). *T. planktonis* was collected only in 3 stations, showing the highest values in the Stations S21 and S23 (71.6 and 75.9 ind. m^−2^, respectively).

**Figure 5:**
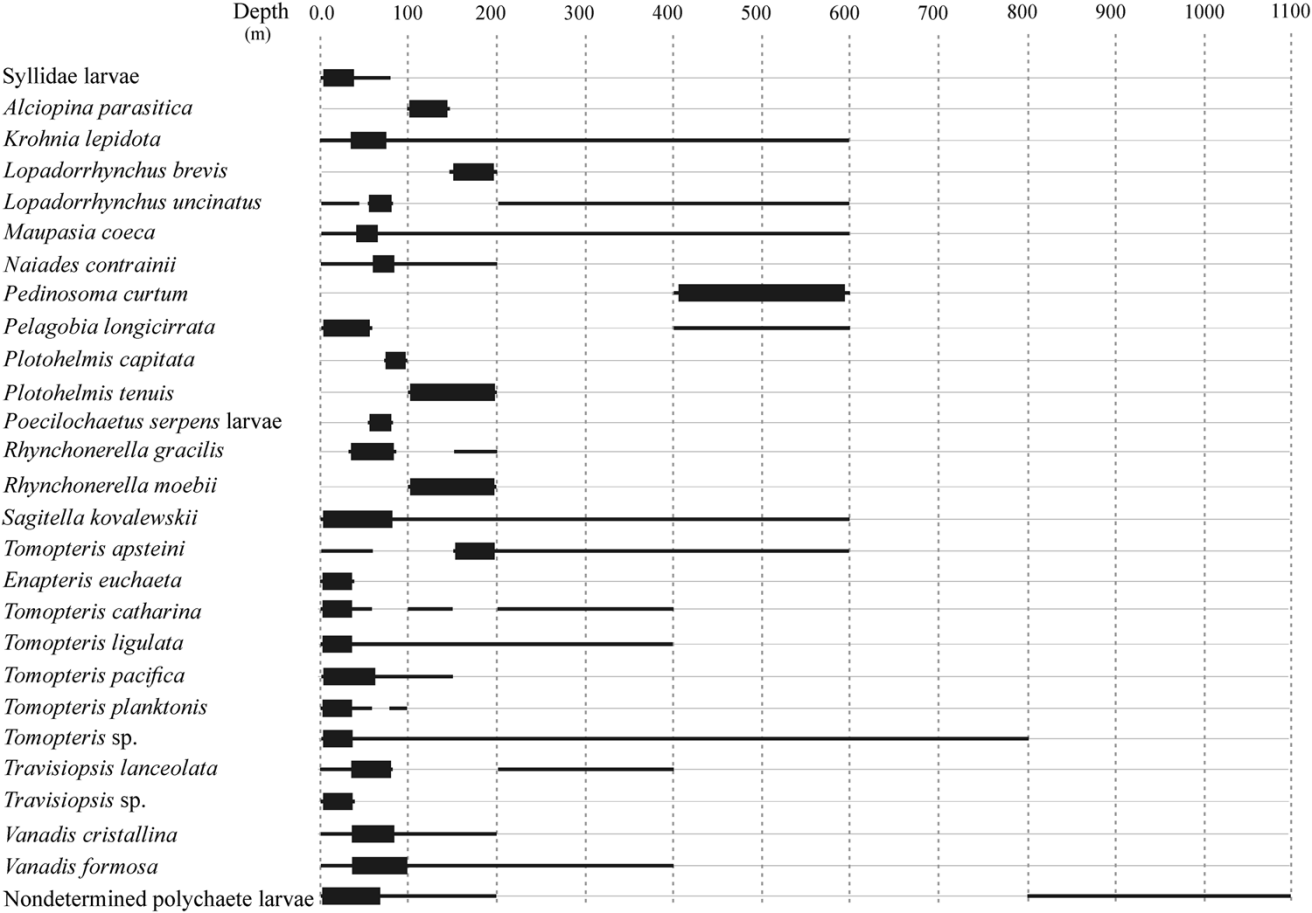
Depth of occurrence and of maximum density for all pelagic polychaete species recorded in the sampling stations.

Vertical range distribution, depth of occurrence and density peak of polychaete species for all sampled stations were shown in Fig. 5. Five species (plus Syllidae larvae) occupy the 0–100 m layer, four species the 0–200 m layer (plus nondetermined polychaete larvae), four species the 0–400 m layer, six species 0–600 m layer, 1 species 0–800 m, 4 species 100–200 m and only *Pedinosoma curtum* the layer 400–600 m. The maximum depth range (0–1100 m) was recorded by undetermined polychaete larvae. Although with different vertical distribution ranges, all species show the maximum density in the epipelagic layer within 200 m, of which about 80% in the 0–100 m layer and only 5 species between 100 and 200 m depth.

#### Relations with the environmental parameters and water mass structure

As expected the polychaete presence was linked to the water mass structure, with a significantly higher density within the samples assigned to ISW respect to ASW, MLIW and ADW (Kruskal-Wallis on medians, p < 0.005) while the comparisons among layers (Surface, DCM, Intermediate, Deep) didn’t highlight significant differences.

**Figure 6:**
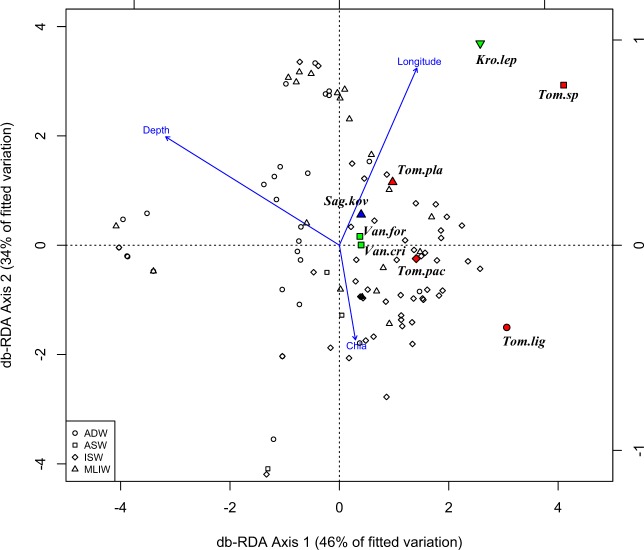
Constrained classifications by distance-based Redundancy Analysis (db-RDA) ordination diagram of the holoplanktonic polychaete species in Southern Adriatic Sea. Blue arrows indicate significant explanatory variables (longitude, depth and chlorophyll *a*). The samples are labeled according to their water-mass cluster membership. The 8 species belonging to the three most abundant genera (90%) are shown: Tomopteridae (red), Alciopidae (green), Typhloscolecidae (blue). Tom.lig: *Tomopteris ligulata*; Tom.sp: *Tomopteris sp*.; Tom.pla: *Tomopteris planktonis*; Tom.pac: *Tomopteris pacifica*; Kro.lep: *Krohnia lepidota*; Van.for: *Vanadis formosa*; Van.cri: *Vanadis crystallina*; Sag.kov.: *Sagitella kovalewskii*.

Figure 6 shows the constrained classification produced by dbRDA analysis. The first two axes (both significant, p < 0.001, ANOVA by axis) explain 87% of the fitted model. Environmental covariates significantly influence the holoplanktonic polichaetes assemblage (Longitude and Depth, p < 0.001; Chlorophyll *a*, p < 0.01, ANOVA by term). The first quarter is associated to the increasing longitude, i.e. towards the southern stations along the Albanian coast, where ISW and MLIW dominate the water column; the species mainly correlated with dbRDA ordination and covariates are in this quarter *Krohnia lepidota* (that dominate in St. S22), *Tomopteris sp*. and *T. planktonis*. The left half plane is mainly associated to an increase of depth (second quarter) and a decrease of longitude (third quarter), corresponding to an increase of latitude, due to the shape of the Adriatic Sea. The stations, mainly the pelagic ones and those along the Italian coast, are characterized by the predominant presence of ADW and MLIW. No species belonging to the three most abundant genera were mapped in this portion of the plane. Finally, the fourth quarter is associated to relatively lower depth and an increase of chlorophyll *a*. The stations mapped here mainly belong to the Surface and DCM layers of the ISW along the northern Albanian coast, that are characterized by the presence of *Tomopteris ligulata* and *T. pacifica*, and to few MLIW. Considering all the sampled stations, polychaete density was positively correlated to temperature and salinity, with significant Spearman’s coefficients (r = 0.37, p < 0.0001 and r = 0.43, p < 0.0001 respectively) whereas a weak correlation was found with chlorophyll *a* concentration (r = 0.11, p = 0.2). Considering the relation between environmental parameters and the six most abundant species separately, only *T. planktonis* abundance was not affected by temperature and salinity, and *Tomopteris* sp. by salinity.

**Figure 7:**
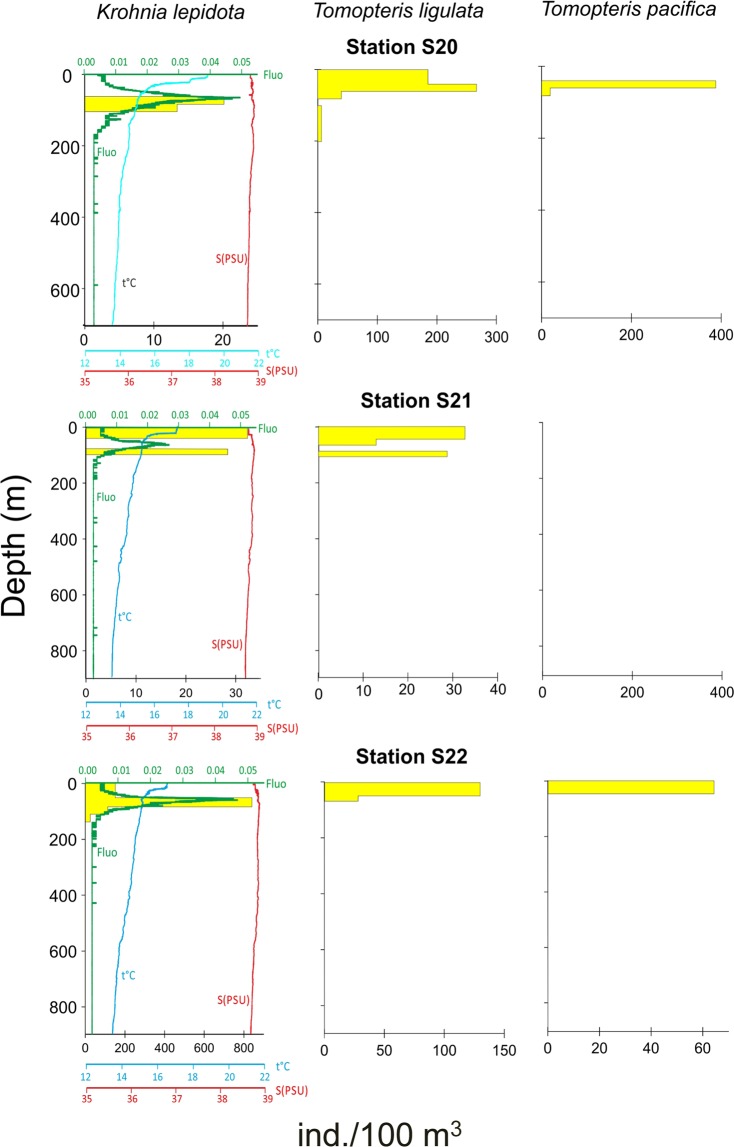
Vertical distribution (ind. 100 m^−3^) of the three most abundant polychaete species and profiles of temperature, salinity and fluorescence collected by BIONESS at the Otranto Channel transect. Note the differences in the density scale. Stations S20 and S22 were sampled during daytime, station S21 was sampled at night.

The interrelationships between the distribution of the three most abundant species (*T. ligulata, K. lepidota, T. pacifica*) and the environmental parameters (temperature, salinity and fluorescence profiles) in the Otranto Channel transect (Sts. S20, S21, S22) are presented in Fig. 7. The three species showed the maximum density below (*K. lepidota)* or within the thermocline (*T. ligulata* and *T. pacifica*) and within the DCM (*K. lepidota*) or just above the DCM (*T. ligulata* and *T. pacifica)*. This last species was not recorded in St. S21. Looking at the day-night vertical distribution of *K. lepidota*, in St. S20 and S22 (diurnal) there is a single subsurface maximum in DCM correspondence, while in St. S21 (nocturnal) the population is divided into two parts (a superficial max and a sub-DCM part).

#### Size distribution of polychaete assemblage

**Figure 8:**
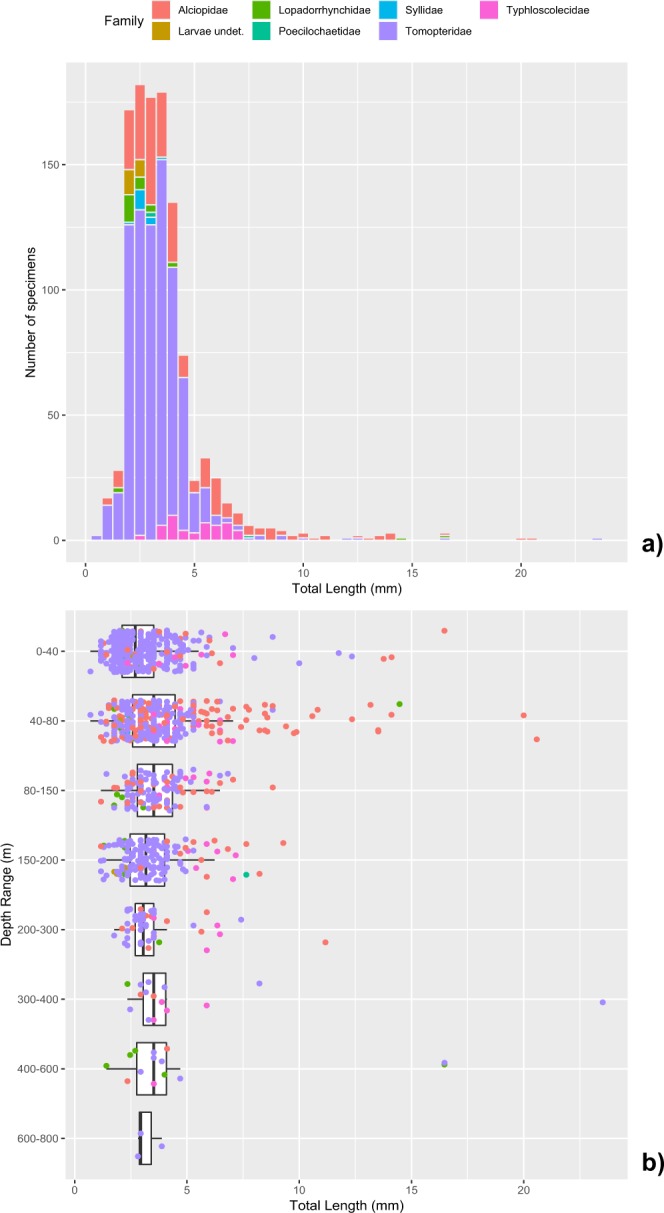
Pelagic polychaetes community collected in Southern Adriatic Sea. (**a**) Size distribution of measured Total Length of the specimens (N = 1154) and (**b**) its dependence on the capture depth range (for each layer, boxplot and medians are indicated; dots represent the single specimens).

The size distribution of the measured total length of the specimens is shown in Fig. 8a. About 75% of the specimens was in the range from 2.0 to 4.5 mm, whereas only 0.5% of the specimens has a total body length beyond the 15.0 mm. The overall shape of the curve indicates a certain degree of bimodality, in particular for the families Alciopidae and Typhloscolecidae, with the first maximum around 2.5–3.0 mm, that can be assigned to juveniles or larval forms (e.g. Syllidae and undetermined taxa) and the second one around 5.5–6.0 mm, possibly related to the adults.

Regarding the individual total lengths (Table 2), *Tomopteris apsteini* showed the largest size range (2.35–23.52 mm) and also the longest specimen (23.52 mm). The smallest individuals belonged to the undefined species of *Tomopteris* genus (0.7 mm), instead the smallest size range (3.76–4.11 mm) belonged to undefined species of *Travisiopsis* genus. The largest mean size (12.35 mm) belonged to *Enapteris euchaeta* and the smallest (2 mm) to *Lopadorrhynchus brevis* Grube, 1855.

**Figure 9:**
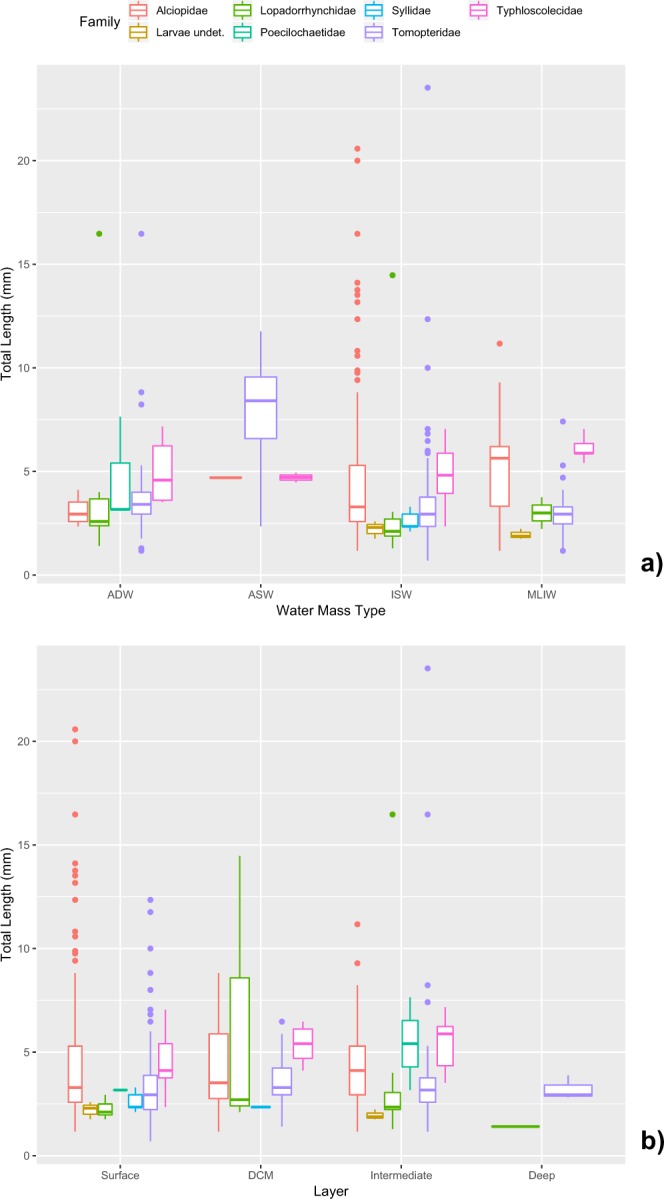
Pelagic polychaetes of the Southern Adriatic Sea. Total Length of the specimens gouped by families versus (**a**) the Water Mass Type and (**b**) the Layer. Boxplot, medians and outliers are indicated.

Specimen size was found to be dependent on the capture depth (Fig. 8b). Overall medians of body lengths were found to be significantly different among the 8 tested layers (ANOVA Kruskal-Wallis, p < 10^–6^). On average, body lengths were relatively higher in the DCM layer (4.17 ± 2.01 mm) than the Surface one (Fisher LSD test, 99% Bonferroni interval) and maxima in the layer 300 to 600 (> 5.1 mm). At surface the lower lengths are due to the predominance of Tomopteridae (*Tomopteris* juveniles) against Alciopidae (*Khronia* and *Vanadis*), that prefer the underlying layer, and to the presence of several larval forms (e.g. Syllidae) in the ISW that largely occupy the surface waters (Fig. 9a,b).

#### Overall and water-mass-related species richness

The sampling effort in the seventeen stations allowed the collection of 146 samples. About 30% of them mainly referring to the Italian shelf and outer shelf did not contain pelagic polychaete specimens at all suggesting a patchy or zonal distribution of this taxonomic group correlated to the oceanographic settings and the circulation patterns. The reference overall sample (sized n = 1154 identified specimens, including 6 singletons and 2 doubletons) exhibited a relevant degree of completeness (99%) well representing the Southern Adriatic pelagic polychaete community in spring. The expected species richness can be estimated to be 25% in excess with respect to the observed one (36 ± 10 species, Chao1 estimator, classic formula). First order jackknife analysis gave an estimated richness of 36 ± 3 species too.

**Figure 10:**
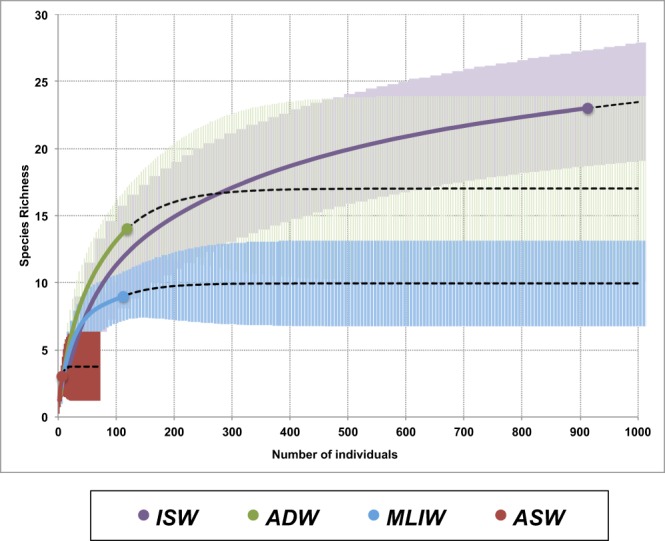
Rarefaction/extrapolation curves of species richness as functions of the number of individuals in the four water types (ISW: Ionian Surface Water; ADW: Adriatic Deep Water; MLIW: Mixed Levantine Intermediate Water; ASW: Adriatic Surface Water). Reference samples are indicated by solid circles, rarefaction by solid lines, extrapolation by dashed lines. Shaded areas indicate the 95% confidence intervals around each curve.

To correctly compare species richness in uneven groups of samples^64,65^, rarefaction/extrapolation curves based on the reference samples in each water mass were plotted (Fig. 10). ISWs exhibit the greatest extrapolated richness, significantly higher than MLIWs (p < 0.05) with which there is no overlap between the 95% confidence intervals. Intermediate value can be assigned to ADW and the lowest to ASW, probably depending also on the extremely uneven sampling in these latter waters. The most relevant difference is that while the extrapolated trend of ISW richness does not show any plateau at a size near the reference sample and far beyond, ADW and MLIW appear to express their asymptotic richness already at 300–400 individuals (17 and 10 species, respectively).

## Discussion

To our knowledge, informations on pelagic polychaete community composition in the South Adriatic Sea are very scarce since available zooplankton data mainly refer to copepods or zooplankton communities^9,33,34,66^. Due to the scarce numerical importance, their species composition is likewise sometimes disregarded in quali-quantitative zooplankton analyses or reported with basic taxonomic detail^67^. Thirteen pelagic polychaete species were reported by Batistić *et al*.^9,33,34^ whereas seventeen species were more recently included in a review dated 2015^37^ (Table 3). Probably as a consequence of the sampling effort (146 samples in a wide geographic area), a higher biodiversity of pelagic polychaetes was found in this study: five species were recorded for the first time in this area out of a total of twenty-two species, in addition to *Tomopteris* sp., *Travisiopsis* sp., Syllidae larval specimens and nondetermined polychaete larvae, with numerical dominance of *T. ligulata, Tomopteris* sp., *K. lepidota*, *T. pacifica*, *V. crystallina* and *T. planktonis*. Hence, to date the overall observed species richness of holopelagic polychaetes within the South Adriatic Sea accounts for twenty-seven species. Interestingly, some species among the ones already recorded^34^ and suggested as possible indicators of the hydroclimatic changes in Southern Adriatic^68^, were not found in this study: *Phalacrophorus pictus*, that is considered a cold species^18^, more commonly found in neritic (2–10 m layer) than pelagic waters^69^, and *Pontodora pelagica* Greeff, 1879 that, conversely, is considered a warm species^70^ and classified as “rare” during winter samplings^69,71^.

Regarding specimen size range, analysis of the size frequency distribution demonstrated a trend in depth distribution by size only for some species. Considering the three most abundant species, *T. ligulata, K. lepidota* and *T. pacifica*, only the first one increased in size with the depth. It should be possible that the older specimens tend to be distributed in deeper layers, instead the smaller specimens inhabit the more superficial layers for trophic reasons^12^. Some hypotheses can be made on the presence of nondetermined larvae found in the deeper pelagic waters. First of all, wind-induced intense cooling at the surface has been already indicated to trigger short-duration open ocean convection events able to locally affect the vertical distribution of zooplankton in Southern Adriatic^34^. Furthermore, larval stages dominate the marine-snow-associated community, with polychaete larvae being one of the most important players in term of biomass^72^. Polychaete larvae can take advantage of the buoyancy of marine snow for their dispersal and utilize marine snow as a transport vehicle and as a food source. This behaviour could justify the abundance of nondetermined larvae found in this study in the pelagic water at 800–1100 m depths.

The comparison of the species richness in the different water masses suggests that ISWs are the main actual and potential carrier of species in the area, though a relevant contribution comes also from ADW that could feed through deep spilling and cascading the deeper basins of the Southern Adriatic with larval or juvenile forms present in the Northern Adriatic, so providing transient windows of remote connectivity near the seabed, especially in late winter-early spring period. Similarly to ISW, the overall species accumulation curve did not reach an asymptote neither within the size of the reference sample nor in the extrapolation to a 5-time size (i.e. 5000 individuals), suggesting a remarkably greater species richness for pelagic polychates in the whole area, in the order of at least 50 species.

During the sampling period, holoplanktonic polychaetes were distributed in both neritic and pelagic waters, although total polychaete density was highest along the Otranto Channel. Species densities were not related to station bottom depth. In fact, the spatial distribution pattern did not change considering only the density data from 0–200 m layer. The number of collected species was lower in the stations on the Italian side respect the Albanian one. This fact has been underlined as a general trend also on microzooplankton^73^, neuston^67^ and fish larvae^74^ and could be the consequence of a lower injection of nutrients and organic material on the Italian side, where rivers are typically absent^46^. In addition to the highest total density, the Otranto Channel waters exhibited also the highest number of species (S20: 16 species; S23: 13 species; S21: 11 species), likely related to the hydrological functioning of this area. A relation between polychaete spatial density and Otranto Channel water circulation can be hypothesized. Due to its morphology the Strait plays in fact a key role in controlling the exchange of water masses and related properties between adjacent basins^75^. Mantziafou & Lascaratos reported the maximum water volume transport in spring and minimum in autumn, with a consequent strong hydrodynamism along the Otranto Channel^76^. Our study was carried out in May, therefore the stations in correspondence of the Otranto Channel were possibly interested by relevant hydrodynamic exchanges that promoted the dragging and accumulation of higher concentration of polychaetes. It was reported that the geographical location of holoplanktonic polychaetes can be correlated to productivity and main water masses^14,77,78^, showing assemblages in areas of high primary and secondary production^66,79–84^. All the species found in the present study (except *Pedinosoma curtum* that represents only the 0.06% of polychaete community) were more abundant in the upper 200 m, and the main part (nineteen of twenty-two species) in the upper 100 m. As already reported, holoplanktonic polychaete species are distributed in the epipelagic region of the water column^5,8,9,71,85,86^, most probably because this layer is characterized by a large supply of food, with highest concentration of phytoplankton, microzooplankton and copepods^87,88^. Surely, the most important gas in water is oxygen, as its role in metabolic processes is essential to all forms of life and it affects the distribution of pelagic organisms at several spatial scales^12^. In the present study, the presence of a maximum of chlorophyll *a* concentration at about 50–80 m allowed us to identify at this layer both a maximum of food availability (phytoplankton) and of oxygen concentration, thus justifying the density of zooplankton and of carnivorous polychaeta in particular. Furthermore pelagic polychaetes were found to be distributed mostly at the thermocline^89^, as observed in this study for the three most abundant species.

Relations between polychaete density and environmental features were investigated. Polychaete distribution patterns were positively correlated with temperature and salinity. Total polychaete density was higher at stations with higher temperature and salinity values, in correspondence of Otranto Channel. The intensity of Eastern Mediterranean influence into the Adriatic depends on the advection of Levantine Intermediate Water, which is controlled by the pressure distribution over the wider area; when the inflow into the Adriatic is strong, one indicator is just the higher salinity^33,90^. High densities of pelagic polychaete were already observed in upwelling regions with high salinity^91^. Vertical polychaete distribution followed the same trend, concentrating in warmer waters in correspondence of or above the thermocline. Pelagic polychaetes distributed mostly at the thermocline (30–100 m), and at the upper and lower Oxygen Minimum Zone (OMZ) interface^89,92^. No relation was found with chlorophyll *a*, surely because the six most abundant species, representing all together more than 80% of the community, belonged to Tomopteridae and Alciopidae families thus showing carnivorous habits^93^ and possibly approaching the phytoplankton-rich layer only in few hours of the day (during vertical migrations). Tomopteridae distribution is worldwide, including oceanic and near-shore waters, from the surface to a few hundred meters depth^1,12^. Feeding habits of Tomopteridae are variable. Some species show a short pharynx and ingest the whole prey or suck out the body fluids, whereas other species miss prey-catching organs and eat microscopic preys^3,12^. Conversely Alciopidae are long, slender and active free-living predators.

Compared with earlier data^9,34,35^, in this study there was a marked change in polychaete dominant species assemblages collected in the open South Adriatic. The species that were abundant in this research (*Tomopteris ligulata, Krohnia lepidota* and *Tomopteris pacifica* (= *T. elegans*)) were absent or poorly represented earlier in spring season (April and May). In particular, *Pelagobia longicirrata* dominated in the whole water colum in April 1993^9^, while no specimens were found in May 1995^35^. The very few specimens of *P. longicirrata* (0.20%) collected in this campaign could confirm the results of Batistić^35^ according to which this species must be considered a cold species. Experimental observations show that the Northern Ionian Gyre (NIG) reverses on multiannual scale^39^. During its anticyclonic phase (e.g. 2006–2010), the Atlantic Water meanders in the northernmost part of the Ionian Sea and induces a general decrease in salinity in the Southern Adriatic^94^. In 2011 the NIG became cyclonic^95^ so favouring the advection of saltier and warmer levantine water in the Southern Adriatic. After a rapid and short inversion in 2012 due to the harsh winter, the NIG circulation returned again to be cyclonic at the beginning of 2013^95^. This scenario is compliant with the dominance of ISW in the Southern Adriatic as observed during the present study and the very low presence of *P. longicirrata* in our samples.

Therefore, in our study the entry of warmer and saltier water favored warm species such as *T. elegans* and non *P. longicirrata* that presents a significant negative correlation with temperature^35^. In conclusion, we agree with Batistić^35^ that these faunal changes can be associated with the change of NIG circulation and, consequently with the periodic modulation of the inflow of Atlantic Water (MAW) or Levantine Water into the Southern Adriatic.

## Supplementary information

Supplementary Table S1
