# Supplementary material for: The Otranto Channel (South Adriatic Sea), a hot-spot area of plankton biodiversity: pelagic polychaetes
Source: Sci Rep. 2019 Dec 20;9:19490. doi: 10.1038/s41598-019-55946-6 (PMC6925135; doi:10.1038/s41598-019-55946-6)
Supplement: Supplementary file 1 — Supplementary Table S1 [file 41598_2019_55946_MOESM1_ESM.pdf]

The Otranto Channel (South Adriatic Sea), a hot-spot area of plankton biodiversity: pelagic polychaetes

ROSANNA GUGLIELMO[1] , ALESSANDRO BERGAMASCO[2*], ROBERTA MINUTOLI[3] , FRANCESCO P. PATTI[1] , GENUARIO BELMONTE[4] , NUNZIACARLA SPANO'[5] , GIACOMO ZAGAMI[3] , VINCENZO BONANZINGA[3] , LETTERIO GUGLIELMO[1] and ANTONIA GRANATA[3]

[1]SZN- Stazione Zoologica Anton Dohrn, Villa Comunale, 80121 Napoli, Italy
[2]CNR-ISMAR (Institute of Marine Sciences, National Research Council), Arsenale - Tesa 104, Castello 2737/F, 30122 Venice, Italy
[3]Dipartimento di Scienze Biologiche, Chimiche, Farmaceutiche ed Ambientali, Università di Messina, 98100 Messina, Italy
[4]CoNISMA O.U. Lecce, DiSTeBA-University of Salento, 73100 Lecce, Italy
[5]Dipartimento di Scienze biomediche, odontoiatriche e delle immagini morfologiche e funzionali, Università di Messina, 98100 Messina, Italy

***Corresponding author**: alessandro.bergamasco@ve.ismar.cnr.it

Station	Hauls Depth Range (m)	FV (m^3)	BIONESS Start time	BIONESS End time	Temperature (°C)	Salinity	Fluorescence (Chl-*a* μg L^{-1})
**S1**	90–80	40.5	20:31:00	20:38:00	12.69	38.64	0.114
	80–70	45.3	20:38:00	20:45:00	12.68	38.62	0.151
	70–60	46.4	20:45:00	20:53:00	12.82	38.64	0.371
	60–50	47.3	20:53:00	21:00:00	12.91	38.64	0.334
	50–40	66.0	21:00:00	21:10:00	13.22	38.65	0.957
	40–30	38.1	21:10:00	21:17:00	13.80	38.72	0.993
	30–20	42.6	21:17:00	21:24:00	14.60	38.75	0.371
	20–10	42.9	21:24:00	21:30:00	16.42	38.31	0.517
	10–0	41.2	21:30:00	21:37:00	19.74	37.73	0.041
**S3**	170–160	45.2	14:12:00	14:22:00	14.16	38.82	0.041
	160–140	50.2	14:22:00	14:32:00	14.97	38.93	0.041
	140–120	50.4	14:32:00	14:42:00	14.99	38.93	0.114
	120–100	54.1	14:42:00	14:54:00	15.05	38.93	0.224
	100–80	66.3	14:54:00	15:04:00	15.19	38.93	0.371
	80–60	59.0	15:04:00	15:14:00	15.35	38.93	0.590
	60–40	61.6	15:14:00	15:25:00	15.75	38.92	0.151
	40–20	50.9	15:25:00	15:34:00	16.85	38.90	0.041
**L41**	550–400	82.1	18:38:00	18:56:00	13.58	38.74	0.004
	400–300	50.5	18:56:00	19:10:00	13.67	38.76	0.004
	300–200	69.7	19:10:00	19:22:00	13.76	38.78	0.004
	200–100	56.6	19:22:00	19:32:00	14.16	38.79	0.261
	100–80	66.3	19:32:00	19:45:00	14.68	38.82	0.371
	80–60	52.2	19:45:00	19:54:00	14.94	38.80	0.334
	60–40	45.1	19:54:00	20:03:00	15.68	38.79	0.114
	40–20	45.7	20:03:00	20:11:00	17.40	38.80	0.078
	0–20	58.3	20:11:00	20:21:00	19.54	38.74	0.114
**S7**	180–160	40.6	15:37:00	15:47:00	15.02	38.94	0.004
	160–140	29.0	15:47:00	15:54:00	15.08	38.94	0.187
	140–120	34.6	15:54:00	16:02:00	15.12	38.94	0.334
	120–100	30.1	16:02:00	16:09:00	15.18	38.93	0.480
	100–80	26.5	16:09:00	16:15:00	15.29	38.93	0.590
	80–60	35.9	16:15:00	16:23:00	15.48	38.91	0.407
	60–40	35.7	16:23:00	16:30:00	16.03	38.93	0.151
	40–20	44.0	16:30:00	16:38:00	17.23	38.89	0.078
	20–0	40.0	16:38:00	16:46:00	21.16	36.63	0.078
**S20**	700–600	27.0	15:09:00	15:18:00	13.75	38.77	0.004
	600–400	59.1	15:18:00	15:30:00	13.97	38.80	0.004
	400–300	65.3	15:30:00	15:44:00	14.10	38.83	0.004
	300–200	45.9	15:44:00	15:53:00	14.54	38.91	0.004
	200–100	52.2	15:53:00	16:05:00	14.92	38.91	0.334
	100–80	35.2	16:05:00	16:14:00	15.04	38.91	0.590
	80–60	34.3	16:14:00	16:22:00	15.24	38.87	0.810
	60–40	29.3	16:22:00	16:30:00	15.66	38.87	0.261
	40–0	43.0	16:30:00	16:41:00	19.10	38.83	0.041
**S21**	900–800	30.2	22:43:00	22:56:00	13.80	38.77	0.004
	800–600	55.1	22:56:00	23:12:00	14.08	38.84	0.004
	600–400	45.2	23:12:00	23:24:00	14.58	38.93	0.004
	400–100	65.2	23:24:00	23:44:00	15.49	38.98	0.078
	100–80	25.6	23:44:00	23:55:00	15.53	38.99	0.261
	80–60	26.7	23:55:00	00:05:00	15.51	38.96	0.590
	60–40	27.8	00:05:00	00:15:00	15.83	38.93	0.187
	40–0	29.0	00:15:00	00:28:00	17.61	38.85	0.114
**S22**	800–900	15.1	18:35:00	18:43:00	13.96	38.80	0.004
	600–800	65.1	18:43:00	19:01:00	14.20	38.85	0.004
	600–400	50.9	19:01:00	19:14:00	14.73	38.93	0.004
	200–400	46.1	19:14:00	19:24:00	15.08	38.93	0.004
	100–200	37.2	19:24:00	19:38:00	15.40	38.97	0.151
	80–100	24.7	19:38:00	19:48:00	15.45	38.97	0.444
	60–80	25.4	19:48:00	19:59:00	15.42	38.93	0.920
	40–60	31.5	19:59:00	20:10:00	15.68	38.93	0.224
	0–40	49.6	20:10:00	20:25:00	16.85	38.83	0.114

Station	Hauls Depth Range (m)	FV (m^3)	BIONESS Start time	BIONESS End time	Temperature (°C)	Salinity	Fluorescence (Chl-*a* µg L^{-1})
**S23**	1100–800	90.4	18:34:00	18:53:00	13.95	38.81	0.004
	800–600	76.6	18:53:00	19:06:00	14.33	38.90	0.004
	600–400	94.0	19:06:00	19:22:00	14.81	38.99	0.004
	400–200	93.5	19:22:00	19:37:00	14.99	38.93	0.004
	200–100	48.0	19:37:00	19:47:00	15.27	38.96	0.041
	100–80	57.4	19:47:00	19:57:00	15.42	38.93	0.590
	80–60	45.1	19:57:00	20:07:00	15.75	38.94	0.407
	60–40	47.2	20:07:00	20:17:00	16.10	38.92	0.114
	40–0	55.1	20:17:00	20:29:00	18.35	38.87	0.078
**S24**	1000–800	53.3	22:39:00	22:54:00	13.81	38.77	0.004
	800–600	65.8	22:54:00	23:07:00	14.07	38.84	0.004
	600–400	83.1	23:07:00	23:22:00	14.47	38.92	0.004
	400–200	74.9	23:22:00	23:34:00	14.77	38.94	0.004
	200–100	40.9	23:34:00	23:41:00	14.92	38.91	0.517
	100–80	45.8	23:41:00	23:50:00	15.09	38.91	0.883
	80–60	49.2	23:50:00	00:00:00	15.49	38.91	0.371
	60–40	51.3	00:00:00	00:10:00	16.27	38.91	0.151
	40–0	89.1	00:10:00	00:23:00	19.31	38.83	0.078
**S25**	210–180	33.9	04:36:00	04:46:00	14.34	38.84	0.114
	180–160	19.5	04:46:00	04:52:00	14.33	38.84	0.114
	160–140	31.6	04:52:00	05:00:00	14.34	38.84	0.187
	140–100	62.2	05:00:00	05:17:00	14.55	38.85	0.444
	100–80	32.5	05:17:00	05:25:00	14.67	38.84	0.664
	80–60	34.5	05:25:00	05:35:00	15.07	38.80	0.480
	60–40	35.4	05:35:00	05:44:00	16.04	38.98	0.151
	40–0	107.0	05:44:00	05:59:00	17.92	39.44	0.004
**S8**	310–250	69.0	04:59:00	05:16:00	14.93	38.94	0.004
	250–200	64.9	05:16:00	05:30:00	15.07	38.94	0.004
	200–150	43.9	05:30:00	05:40:00	15.14	38.93	0.004
	150–100	59.2	05:40:00	05:52:00	15.15	38.92	0.078
	100–80	38.5	05:52:00	06:00:00	15.23	38.93	0.297
	80–60	36.5	06:00:00	06:07:00	15.39	38.93	1.360
	60–40	37.4	06:07:00	06:15:00	15.80	38.91	0.151
	40–20	30.3	06:15:00	06:22:00	16.82	38.92	0.151
	20–0	29.3	06:22:00	06:35:00	20.21	38.73	0.151
**S10**	1096–800	77.3	00:26:00	00:41:00	13.63	38.77	0.004
	800–600	72.5	00:41:00	00:53:00	13.90	38.79	0.004
	600–400	79.6	00:53:00	01:05:00	13.98	38.81	0.004
	400–200	89.1	01:05:00	01:20:00	13.98	38.82	0.004
	200–100	68.7	01:20:00	01:34:00	13.99	38.82	0.444
	100–80	38.8	01:34:00	01:42:00	14.16	38.83	0.664
	80–60	45.6	01:42:00	01:51:00	14.30	38.83	0.883
	60–40	38.7	01:51:00	01:58:00	14.63	38.82	0.151
	40–0	39.9	01:58:00	02:07:00	20.16	38.80	0.078
**S15**	900–800	24.9	06:01:00	06:09:00	13.37	38.74	0.004
	800–600	105.4	06:09:00	06:28:00	13.62	38.76	0.004
	400–200	74.6	06:28:00	06:44:00	13.97	38.81	0.004
	200–100	49.0	06:53:00	07:01:00	14.59	38.88	0.151
	100–80	60.7	07:01:00	07:11:00	14.57	38.85	0.334
	80–60	47.5	07:11:00	07:19:00	14.92	38.83	0.334
	60–40	47.0	07:19:00	07:28:00	15.73	38.89	0.224
	40–0	57.1	07:28:00	07:38:00	19.42	38.71	0.004
**S16c**	300–250	30,75	11:58:00	12:05:00	14.80	38.93	0.004
	250–200	59,63	12:05:00	12:16:00	14.92	38.94	0.004
	200–150	48,00	12:16:00	12:24:00	14.94	38.92	0.004
	150–100	40,42	12:24:00	12:40:00	15.12	38.93	0.151
	100–80	40,24	12:40:00	12:47:00	15.14	38.92	0.517
	80–60	35,98	12:47:00	12:55:00	15.48	38.93	0.627
	60–40	29,86	12:55:00	12:55:00	15.79	38.92	0.151
	0–40	35,72	12:55:00	13:00:00	20.49	38.66	0.041
**S19**	100–90	48.1	10:23:00	10:34:00	14.68	38.88	0.261
	90–80	42.4	10:34:00	10:42:00	14.72	38.88	0.297

Station	Hauls Depth Range (m)	FV (m^3)	BIONESS Start time	End time	Temperature (°C)	Salinity	Fluorescence (Chl-*a* µg L^{-1})
	80–70	49.3	10:42:00	10:51:00	14.83	38.89	0.444
	40–30	67.2	10:51:00	11:14:00	15.79	38.84	0.224
	30–20	48.3	11:14:00	11:23:00	18.76	38.42	0.297
	20–10	25.8	11:23:00	11:31:00	18.97	38.21	0.114
	10–0	32.7	11:31:00	11:42:00	19.08	38.17	0.032
**S14**	600–400	57.8	18:18:00	18:33:00	13.91	38.80	0.004
	400–300	57.9	18:33:00	18:44:00	13.97	38.81	0.004
	300–200	60.4	18:44:00	18:56:00	13.94	38.78	0.078
	200–100	45.9	18:56:00	19:05:00	14.69	38.88	0.261
	100–80	34.8	19:05:00	19:14:00	14.92	38.91	0.517
	80–60	34.7	19:14:00	19:22:00	15.06	38.92	0.627
	60–40	32.6	19:22:00	19:31:00	15.29	38.89	0.371
	40–20	49.2	19:31:00	19:41:00	18.25	38.76	0.151
	20–0	46.3	19:41:00	18:51:00	18.88	38.54	0.224
**S11**	1060–800	47.8	07:45:00	07:56:00	13.39	38.72	0.004
	800–600	79.9	07:56:00	08:09:00	13.74	38.76	0.004
	600–400	90.2	08:09:00	08:24:00	13.97	38.81	0.004
	400–200	92.3	08:24:00	08:36:00	13.98	38.81	0.004
	200–100	62.2	08:36:00	08:46:00	14.00	38.82	0.114
	100–80	63.3	08:46:00	08:56:00	14.05	38.82	0.737
	80–60	63.7	08:56:00	09:06:00	14.28	38.82	0.517
	60–40	58.2	09:06:00	09:15:00	14.64	38.82	0.187
	40–0	94.7	09:15:00	09:25:00	18.41	38.80	0.032

Tab.S1 Sampled strata, filtered volumes, BIONESS tow times, temperature, salinity and fluorescence data. A maximum of 9 depth intervals were sampled. Samples were collected both during daytime (Sts. L41, S3, S7, S8, S16c, S15, S22, S23, S20, S19, S14, S11) and nighttime (Sts.S1, S10, S21, S24, S25). During the oceanographic cruise: local sunrise 05:30, local sunset 20:00.
